# Continuous Renal Replacement Therapy for Patients With Sepsis in a Low-Resource Medical Intensive Care Unit (MICU): Incidence, Risk Factors, and Outcomes

**DOI:** 10.7759/cureus.103319

**Published:** 2026-02-09

**Authors:** Milka Jandric, Danica Momcicevic, Sasa Dragic, Biljana Zlojutro, Tijana Kovacevic, Goran Baric, Boris Tomic, Sanja Davogic, Jovana Malic, Pedja Kovacevic

**Affiliations:** 1 Medical Intensive Care Unit, University Clinical Centre of the Republic of Srpska, Banja Luka, BIH; 2 Faculty of Medicine, University of Banja Luka, Banja Luka, BIH; 3 Department of Pharmacy, University Clinical Centre of the Republic of Srpska, Banja Luka, BIH

**Keywords:** critically ill patients, intensive care unit, renal replacement therapy, sepsis, sepsis-associated acute kidney injury

## Abstract

Introduction

Sepsis is one of the leading causes of ICU admissions, with a substantial proportion of patients developing sepsis-associated acute kidney injury (S-AKI). In such cases, continuous renal replacement therapy (CRRT) is a cornerstone of renal supportive care; however, evidence from low-resource settings (LRS) remains limited. This study aimed to describe the demographic and clinical characteristics of patients with S-AKI treated with CRRT in an LRS medical ICU (MICU), as well as to identify predictors of mortality.

Subjects and methods

This retrospective, observational, consecutive, single-center study included adult patients admitted to the MICU between June 1, 2023, and June 1, 2024, with a diagnosis of sepsis complicated by S-AKI, and managed with CRRT. Statistical analyses were conducted using the Mann-Whitney U and Pearson χ² tests, and multinomial logistic regression was used to identify independent predictors of mortality. ORs were reported for key predictors, and Kaplan-Meier survival analysis was performed to assess time to event.

Results

A total of 96 patients with S-AKI were treated with CRRT (65 male patients, median age 64.5 years). The 28-day all-cause mortality rate was 69.8%, with a high rate of septic shock at admission among nonsurvivors (n=49, p= 0.019). Kaplan-Meier analysis demonstrated a median survival of 12 days (95%CI: 8.95-15.05). The majority of patients were admitted from hospital wards (n=59), and the most common comorbidities were hypertension (n=63), diabetes (n=38), and cardiomyopathy (n=27). Survivors had a longer MICU length of stay (p= 0.003). Nonsurvivors had a higher initial Sequential Organ Failure Assessment and Simplified Acute Physiology Score II (SAPS II) scores at MICU admission (p< 0.000), and prominent abnormalities at CRRT initiation for albumin (p= 0.004), troponine I (p= 0.032), and lactate (p= 0.004). Invasive mechanical ventilation and vasopressor therapy were predominantly used among nonsurvivors (p< 0.001). Continuous Venovenous Hemodiafiltration (CVVHDF) was the CRRT modality used for all patients (three patients used a combination of continuous and intermittent techniques), and hemoadsorption filters were used in 38 patients. The preferential indications for CVVHDF included anuria and profound metabolic acidosis, either in combination (n=51) or alone (n=17 and n=22). The most common sources of sepsis were pneumonia (n=42), urinary tract infection (n=13), multiple site infection (n=11), and abdomen (n=11). At MICU admission, blood cultures were positive in 34 patients (21 with gram-positive bacteria), urine cultures in 21 patients (12 with gram-negative bacteria), and tracheal aspirate/bronchoalveolar lavage in 39 patients (26 with gram-negative bacteria). Among the patients, 10 had a concurrent viral infection, six had candidiasis, and three had aspergillosis. Logistic regression identified an association between poor outcome and SAPS II at MICU admission (OR=1.07; 95%CI: 1.03-1.12), albumin (OR=0.89; 95%CI: 0.81-5.63), and vasopressor therapy (OR= 8.36; 95%CI: 1.51-46.33) at CRRT initiation.

Conclusion

Patients with S-AKI requiring CRRT represent a particularly vulnerable subgroup with a high risk of poor outcomes, especially when presenting with septic shock. In this single-center, low-resource MICU study, independent predictors of mortality were a high SAPS II score at MICU admission, as well as vasopressor requirement and hypoalbuminemia at CRRT initiation.

## Introduction

Sepsis is a critical clinical entity that remains one of the leading causes of morbidity and mortality in intensive care units (ICUs) worldwide. In 2017, it was estimated to account for 49 million cases and 11 million deaths globally. Strikingly, 85% of these cases and deaths occurred in low-resource settings (LRS), often affecting vulnerable and underserved populations [1]. Vincent et al. reported that this patient population experiences more severe organ dysfunction, longer hospital stays, and higher mortality rates [2].

Upon ICU admission, approximately 30% of critically ill patients have sepsis, while about 10.4% present with septic shock, which is associated with a 30-day mortality rate of approximately 35% [3-5]. On the other hand, this percentage is likely even higher in LRS, although a major challenge lies in the limited availability of data from these regions [6,7]. Sepsis-associated acute kidney injury (S-AKI), defined as a sudden deterioration in renal function in the presence of sepsis, is an early, common, life-threatening complication and an independent risk factor for mortality [8].

The pathophysiology of S-AKI is complex and multifactorial, involving dysregulated immune responses with complement activation, hemodynamic alterations with predominant microcirculatory dysfunction, endothelial injury with increased microvascular permeability, and tubular epithelial cell injury and dysfunction. The diagnosis is based on the presence of both sepsis and acute kidney injury (AKI), with AKI defined by the KDIGO (Kidney Disease: Improving Global Outcomes) criteria [9].

Nearly half of all septic patients develop S-AKI, and one in four die during hospitalization, while the presence of septic shock significantly worsens outcomes [10]. Renal replacement therapy (RRT), a key form of renal supportive care, is used in 23.5% of AKI patients according to the Acute Kidney Injury-Epidemiologic Prospective Investigation (AKI-EPI) 1 study, most commonly in the form of continuous renal replacement therapy (CRRT) [11]. In certain situations, clinicians may consider the use of hemoadsorptive filters; however, according to the 2021 Surviving Sepsis Campaign guidelines, there is insufficient evidence to support a recommendation for their routine use [12].

Although the large AKI-EPI 1 study was conducted, data on S-AKI in critically ill patients from LRS remain limited. Possible explanations for this are limited diagnostic and research infrastructure, lack of registries, limited access to the ICU, a shortage of personnel dedicated to research activities, and financial barriers to publishing in scientific journals. The aim of this study was to identify predictors of mortality and describe demographic and clinical characteristics of patients with S-AKI treated with CRRT in a medical ICU (MICU) in Bosnia and Herzegovina.

## Materials and methods

This was a retrospective, observational, consecutive, single-center study conducted at the University Clinical Centre of the Republic of Srpska in Banja Luka, Bosnia and Herzegovina. The study was approved by the Ethics Committee of the University Clinical Centre of the Republic of Srpska (approval number: 01-19-495-2/24, dated November 28, 2024), and due to the observational nature of the study, informed consent was waived for all patients.

Study population

Inclusion criteria were adult patients (aged > 18 years) who were admitted to the MICU between June 1, 2023, and June 1, 2024, due to sepsis and S-AKI, with clinical and laboratory evidence of sepsis or septic shock, and the requirement for CRRT. Exclusion criteria included shock of other origin, end-stage renal disease requiring dialysis, pregnancy, and death within 24 hours of MICU admission.

**Figure 1:**
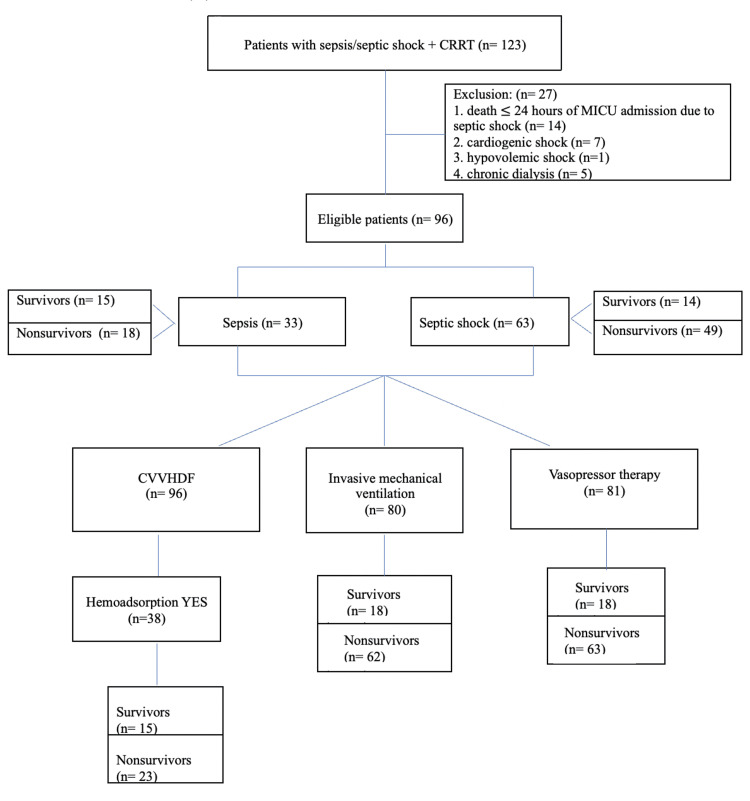
Flow chart of the study CVVHDF: continuous venovenous hemodiafiltration; CRRT: continuous renal replacement therapy

The sample consisted of 96 eligible patients. All of them were managed with CRRT. Of the initially 123 patients, 27 were excluded for the following reasons: death within 24 hours of MICU admission due to septic shock (n=14), cardiogenic shock (n=7), hypovolemic shock (n=1), and pre-existing chronic dialysis (n=5). The flow chart of the study is presented in Figure 1. 

Definitions

According to the Sepsis-3 guidelines [13], sepsis was diagnosed based on suspected or documented infection and organ dysfunction, defined as an acute increase of ≥2 points in the Sequential Organ Failure Assessment (SOFA) score, whereas septic shock was diagnosed in patients with sepsis who required vasopressors to maintain a mean arterial pressure ≥65 mmHg and had a serum lactate level >2 mmol/L despite adequate fluid resuscitation.

S-AKI was defined as acute kidney injury that develops in the context of sepsis, characterized by an abrupt deterioration of renal function, manifested by an increase in serum creatinine and/or a decrease in urine output. The indication for CRRT initiation was assessed based on urine output and acid-base imbalance: oliguria/anuria with fluid overload and with/without electrolyte disturbances, and severe metabolic acidosis (pH < 7.1), either in combination or alone. 

Procedures

All patients were treated with CRRT in accordance with the local protocol for septic patients. In addition to nurses’ familiarity with this procedure, intermittent hemodialysis has technical limitations, as patients must be transported to another location, which represents a risk for critically ill patients. The CRRT dose was 25-30 mL/kg/h, CVVHDF was the modality used, and the most commonly used ratio of dialysate to replacement fluid was 1:1.

CRRT was discontinued upon renal recovery or patient death. A subset of patients additionally received hemoadsorptive therapy (CytoSorb (CytoSorbents Corporation, Princeton, New Jersey, United States), Oxyris (Vantive Health LLC, Deerfield, Illinois, United States), or a combination of both), based on filter availability and inflammatory markers results. The indication for hemoadsorption was determined by high vasopressor requirement and elevated inflammatory markers. Our center does not have clear cut-off values for hemoadsorption, and the decision is based on a combination of high vasopressor requirements (norepinephrine typically >0.4-0.5 mcg/kg/minute), increased interleukin-6 (>300-400 pg/mL), and elevated procalcitonin (PCT) (> 2 ng/mL). The term "multiple-site infection" refers to the presence of infections at more than one anatomical site at admission (e.g., pneumonia + urinary infection, abdominal + urinary infection).

Outcomes

The primary outcome of the study was 28-day all-cause mortality, determined by review of the hospital information system on day 28. Baseline demographic and clinical characteristics were described, and associations between mortality and CRRT characteristics, hemoadsorptive therapy, and sepsis characteristics were explored as secondary analyses.

Data collection

The data collected and analyzed included age, sex, comorbidities, type of admission, presence of septic shock at MICU admission, length of hospital stay prior to MICU admission, SOFA score, and Simplified Acute Physiology Score II (SAPS II) within 24 hours after MICU admission. Venous blood samples were collected for laboratory analyses on the day of CRRT initiation, analyzed, and compared between survivors and nonsurvivors for white blood cell count, platelet count, hemoglobin, C-reactive protein, procalcitonin, interleukin-6, albumin, creatinine, urea, D-dimer, troponin I, and lactate. Additional data included the need for endotracheal intubation and mechanical ventilation, vasopressor therapy, details of CRRT (modality, day of initiation, indications, type of anticoagulation, and use and type of hemoadsorption filter), length of stay in the MICU, source of sepsis, isolated pathogens from blood, urine, and tracheal aspirate cultures, the presence of viral infections, and aspergillosis. After data collection from hospital medical records, patients were stratified into survivors and nonsurvivors (mortality was based on death certificates), and clinical and treatment-related variables were compared between groups.

Data analysis

Data analysis was performed using the IBM SPSS Statistics for Windows, version 26.0 (Released 2019, IBM Corp., Armonk, New York, United States). Some missing data were not imputed, and analyses were conducted using available cases. Continuous variables were summarized as medians and interquartile ranges (IQR), while categorical variables were presented as frequencies and percentages. Normality of data was tested using Sthe hapiro-Wilk and Kolmogorov-Smirnov tests. The Mann-Whitney U test was used to compare the non-normally distributed continuous variables, while Pearson's χ2 test was used for categorical variables. Multinomial logistic regression and analysis were performed in order to determine the causality of independent predictors of mortality. Kaplan-Meier survivor analysis was conducted to assess time to event. A value of p≤ 0.05 was considered statistically significant.

## Results

**Figure 2:**
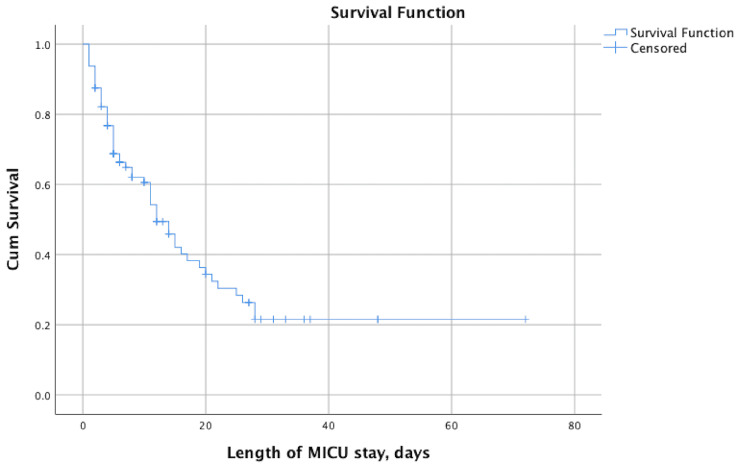
The Kaplan-Meier curve for mortality showing a median survival of 12 days (95%CI 8.950-15.050) MICU: medical intensive care unit

Over a one-year period, 96 patients who were admitted to MICU due to sepsis and S-AKI were treated with CRRT. The 28-day all-cause mortality rate was 69.8%. The median age was 64.5 years, and 67.7% of them were male. Patients were admitted to the MICU from other hospital wards (61.5%), the emergency department (26%), or regional hospitals 12 (12.5%). The median hospital stay prior to MICU admission was two days, and the median length of MICU stay was 6.5 days, longer in survivors (p= 0.003). Kaplan-Meier analysis demonstrated a median survival of 12 days (95%CI: 8.95-15.05) (Figure 2). The most common comorbidities were hypertension (65.6%), diabetes (39.6%), cardiomyopathy (28.1%), anemia (19.8%), chronic kidney disease (18.8%), malignancy (16.7%), chronic obstructive pulmonary disease (14.6%), and liver disease (7.3%). The median SOFA score was 11, and the median SAPS II score was 49, both significantly higher in nonsurvivors (p< 0.001). Septic shock was present in 65.6% of patients upon MICU admission, predominantly in nonsurvivors (p= 0.019). Demographic characteristics of patients are presented in Table 1.

**Table 1:** Baseline characteristics of patients with sepsis treated with CRRT in the MICU Asterisks indicate statistical significance: p≤ 0.05; Statistical tests used: Mann-Whitney U test, Pearson's χ2 test SOFA: Sequential Organ Failure Assessment Score; SAPS II: Simplified Acute Physiology Score II; MICU: Medical Intensive Care Unit; CRRT: continuous renal replacement therapy; MICU: medical intensive care unit

Parameters	All (n= 96)	Survivors (n= 29)	Nonsurvivors (n= 67)	p-value
Age (years), median (IQR)	64.50 (20.75)	62 (23)	67 (20)	0.059
Sex (male), n (%)	65 (67.70%)	19 (29.20%)	46 (70.80%)	0.116
Type of admission, n (%)				
Emergency department	25 (26%)	8 (32%)	17 (68%)	0.057
Hospital ward	59 (61.50%)	14 (23.70%)	45 (76.30%)	
Regional hospital	12 (12.50%)	7 (58.30%)	5 (41.70%)	
SOFA score, median (IQR)	11 (4)	9 (4)	11 (4)	< 0.001*
SAPS II score, median (IQR)	49 (23.50)	37 (17)	56 (23)	< 0.001*
Comorbidities, n (%)				
Hypertension	63 (65.60%)	19 (30.20%)	44 (69.80%)	0.988
Diabetes	38 (39.60%)	11 (28.90%)	27 (71.10%)	0.828
Cardiomyopathy	27 (28.10%)	8 (29.60%)	19 (70.40%)	0.938
Anemia	19 (19.80%)	6 (31.60%)	13 (68.40%)	0.884
Chronic kidney disease	18 (18.80%)	8 (44.40%)	10 (55.60%)	0.144
Cancer	16 (16.70%)	3 (18.80%)	13 (81.30%)	0.274
Chronic obstructive pulmonary disease	14 (14.60%)	4 (28.60%)	10 (71.40%)	0.885
Liver disease	7 (7.30%)	0	7 (100%)	0.071
Septic shock at MICU admission, n (%)	63 (65.60%)	14 (22.20%)	49 (77.80%)	0.019*
Hospital stay prior to MICU admission (days), median (IQR)	2 (10)	1 (8)	2 (7)	0.444
Length of MICU stay (days), median (IQR)	6.50 (10.75)	10 (23)	6 (10)	0.003*

On the day of CRRT initiation, there were significant differences between survivors and nonsurvivors in albumin (p= 0.004), troponin I (p= 0.032), and lactate (p=0.004), with more abnormal values observed in nonsurvivors. Invasive mechanical ventilation and vasopressors were predominantly used among nonsurvivors (p< 0.001). CVVHDF was the CRRT modality used for all patients, while three patients used a combination of continuous and intermittent techniques (p= 0.007). The preferential indications for CVVHDF included oliguria/anuria and profound metabolic acidosis, either in combination (51 patients) or alone (17 patients and 22 patients, respectively). The most commonly used anticoagulant therapy for CRRT was unfractionated heparin (61.5% of patients) and regional citrate anticoagulation (19.8% of patients). Hemoadsorption filters were used in 40.62% of patients (p= 0.057), and the most commonly used filter was CytoSorb (31.3%). CRRT was initiated on day 1 of MICU admission, with an average of one CRRT set used per patient. There was no significant difference in the number of CRRT and the number of hemoadsorption filters used in survivors and nonsurvivors (p= 0.893 and p= 0.330). Clinical data during the MICU stay are presented in Table 2. 

**Table 2:** Laboratory and clinical data of septic patients treated with CRRT Asterisks indicate statistical significance: p ≤ 0.05; Statistical test used: Mann-Whitney U test; Pearson's χ2 test RV: reference value; CRRT: continuous renal replacement therapy; WBC: white blood cells; PLT: platelets; Hb: hemoglobin; CRP: C-reactive protein; PCT: procalcitonin; CVVHDF: continuous veno-venous hemodiafiltration; IHD: intermittent hemodialysis; MICU: medical intensive care unit; UFH: unfractionated heparin; RCA: regional citrate anticoagulation

Parameters	All (n= 96)	Survivors (n= 29)	Nonsurvivors (n= 67)	p-value
Laboratory test at CRRT initiation, median (IQR)				
WBC (RV: 3.40-9.79 (1e9)/L)	14.80 (13.08)	14.46 (12)	14.02 (12)	0.735
PLT (RV: 158-424 (1e9)/L)	157 (158.25)	185 (166)	143 (168)	0.083
Hb (RV: 158-424 g/L)	107.50 (35.25)	112.50 (37)	106 (32)	0.687
CRP (RV: 0-5 mg/L)	146.95 (186)	154.15 (238)	148.40 (174)	0.353
PCT (RV: < 0.5 ng/mL)	6.07 (16.82)	5.83 (17)	5.93 (15)	0.780
Albumin (RV: 35-52 g/L)	27 (7)	28.50 (7)	24 (8)	0.004*
Creatinine (RV: 45-84 µmol/L)	247.50 (348.75)	305.50 (475)	241 (303)	0.808
Urea (RV: 2.8-7.2 mmol/L)	20.80 (17.85)	19.15 (22)	21.60 (16)	0.150
D-dimer (RV: 0.00-0.50 mg/L)	5.62 (8.94)	9.58 (11)	9.01 (14)	0.316
Troponin I (RV: 0-34.2 pg/mL)	69.30 (402.75)	13.70 (89)	61.80 (456)	0.032*
Interleukin-6 (RV: 0-7 pg/mL)	500 (1092.75)	363 (750)	581 (3601)	0.561
Lactate (RV: 0.00-2.0 mmol/L)	3.20 (4.28)	1.99 (4)	4.95 (9)	0.004*
Intubation and invasive mechanical ventilation, n (%)	80 (83.30%)	18 (22.50%)	62 (77.50%)	< 0.001*
Vasopressor therapy, n (%)	81 (84.40%)	18 (22.20%)	63 (77.80%)	< 0.001*
Type of CRRT, n (%)				
CVVHDF	93 (96.90%)	26 (28%)	67 (72%)	0.007*
CVVHDF + IHD	3 (3.10%)	3 (100%)	0	
Indication for CRRT, n (%)				
Metabolic acidosis	24 (25%)	10 (41.70%)	14 (58.30%)	0.274
Oliguria/Anuria	15 (15.62%)	3 (20%)	12 (80%)	
Acidosis+Oliguria/Anuria	51 (53.12%)	13 (25.50%)	38 (74.50%)	
Hemoadsorption	6 (6.25%)	3 (50%)	3 (50%)	
Anticoagulation, n (%)				
UFH	59 (61.50%)	21 (35.60%)	38 (64.40%)	0.093
RCA	19 (19.80%)	7 (36.8%)	12 (63.20%)	
UFH + RCA	1 (1%)	0	1 (100%)	
None	17 (17.70%)	1 (5.90%)	16 (94.10%)	
Day of MICU stay at CRRT initiation, median (IQR)	1 (2)	1 (2)	1 (2)	0.523
CRRT sets per patient, median (IQR)	1 (2)	2 (2)	1 (2)	0.893
Hemoadsorption, n (%)	38 (39.58%)	15 (39.50%)	23 (60.50%)	0.117
Hemoadsoption filter, n (%)				
CytoSorb	30 (31.30%)	12 (24.10%)	18 (60%)	0.057
Oxiris	2 (2.10%)	2 (100%)	0	
CytoSorb + Oxiris	6 (6.30%)	1 (16.70%)	5 (83.30%)	
None	58 (60.40%)	14 (24.10%)	44 (75.90%)	
Hemoadsoption filters per patient, median (IQR)	0 (1)	0 (1)	0 (1)	0.330

The most prevalent source of sepsis was pneumonia (n=42), urinary tract infection (n=13), multiple site infection (n=11), and abdomen (n=11). At MICU admission, blood cultures were positive in 34 patients (gram-positive bacteria were isolated from 21 patients), urine cultures in 21 patients (gram-negative bacteria were isolated from 12 patients), and tracheal aspirate/bronchoalveolar lavage (BAL) in 39 patients (gram-negative bacteria were isolated from 26 patients). Among the patients, 10 had a concurrent viral infection, and three had aspergillosis. Data regarding the sources of sepsis and culture results are presented in Table 3.

**Table 3:** Sepsis orgin and cultures results in septic patients treated with CRRT Asterisks indicate statistical significance: p ≤ 0.05; Statistical test were used: Mann-Whitney U test, Pearson's χ2 test MICU: medical iIntensive care unit; BAL: bronchoalveolar lavage; CRRT: continuous renal replacement therapy

Parameters	All (n=96)	Survivors (n=29)	Nonsurvivors (n=67)	p-value
Source of sepsis, n (%)				
Respiratory system	42 (43.75%)	11 (26.20%)	31 (73.10%)	0.862
Urinary system	13 (13.54%)	3 (23.10%)	10 (76.90%)	
Multiple infection	11 (11.46%)	4 (36.40%)	7 (63.60%)	
Abdomen	11 (11.46%)	5 (45.50%)	6 (54.50%)	
Bloodstream	7 (7.29%)	2 (28.60%)	5 (71.40%)	
Skin	7 (7.29%)	2 (28.60%)	5 (71.40%)	
Unknown	4 (4.17%)	2 (50%)	2 (50%)	
Central nervous system	1 (1.04%)	0	1 (100%)	
Blood culture at MICU admission, n (%)				
Sterile	62 (64.58%)	19 (30.60%)	43 (69.40%)	0.659
Gram-positive bacteria	21 (21.87%)	5 (23.80%)	16 (76.20%)	
Gram-negative bacteria	13 (13.54%)	5 (38.50%)	8 (61.50%)	
Urine culture at MICU admission, n (%)				
Sterile	70 (72.92%)	24 (34.30%)	46 (65.70%)	0.345
Gram-positive bacteria	7 (7.29%)	1 (14.30%)	6 (85.70%)	
Gram-negative bacteria	12 (12.50%)	4 (33.30%)	8 (66.70%)	
*Candida* spp.	2 (2.08%)	0	2 (100%)	
Not sampled	5 (5.21%)	0	5 (100%)	
Tracheal aspirate/BAL at MICU admission, n (%)				
Physio phlora	42 (43.75%)	8 (19%)	34 (81%)	0.002*
Gram-positive bacteria	9 (9.37%)	2 (22.20%)	7 (77.80%)	
Gram-negative bacteria	26 (27.08%)	6 (23.10%)	20 (76.90%)	
Not sampled	15 (15.62%)	11 (73.30%)	4 (26.70%)	
*Candida* spp.	4 (4.17%)	2 (50%)	2 (50%)	
Viral disease at MICU admission, n (%)	10 (10.42%)	1 (10%)	9 (90%)	0.141
Aspergillosis at MICU admission, n (%)	3 (3.12%)	0	3 (100%)	0.247

**Figure 3:**
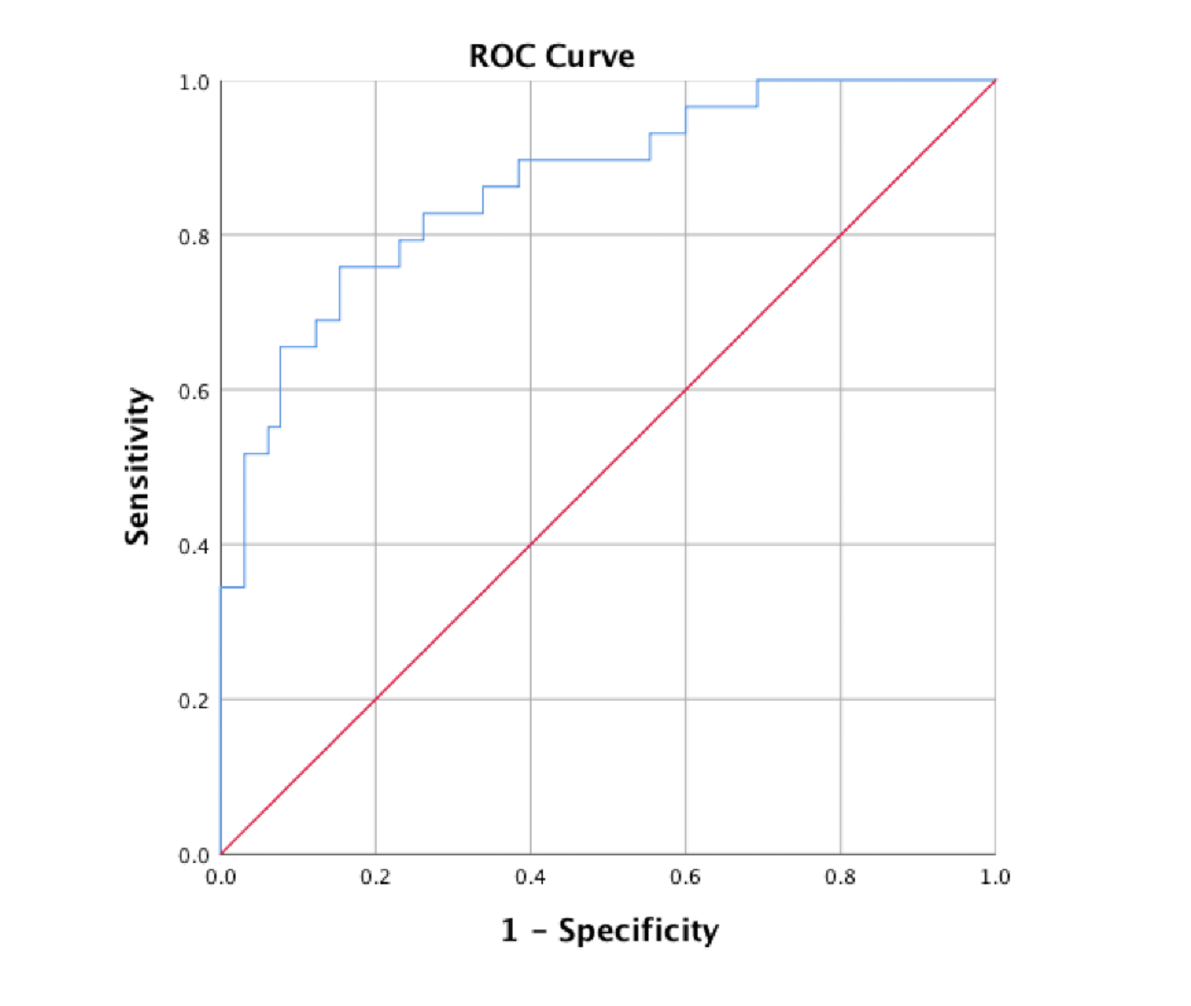
ROC curve for the logistic regression model AUC= 0.864 (CI 95% 0.783-0.945; p< 0.001) ROC: receiver operating characteristic; AUC: area under the curve

In logistic regression analysis, a significant association was identified between poor outcome and SAPS II at MICU admission (OR=1.07; 95%CI: 1.03-1.12), need for vasopressor therapy (OR= 8.36; 95%CI: 1.51-46.33), and hypoalbuminemia at the CRRT initiation (OR=0.89; 95%CI: 0.81-5.63). The area under the receiver operating characteristic curve (AUROC) for the logistic regression model was 0.864 (95%CI 0.783-0.945; p< 0.001). Results of this analysis are presented in Table 4 and Figure 3. 

**Table 4:** Results of logistic regression analysis for mortality Asterisks indicate statistical significance: p ≤ 0.05. OR: odds ratio; CI: confidence interval; SAPS II: Simplified Acute Physiology Score II

Parameter	p-value	OR	95% CI
SAPS II	0.002*	1.074	1.027-1.124
Male	0.963	0.971	0.278-3.397
Liver disease	0.999	0	0
Albumin	0.015*	0.893	0.815-5.632
Need for vasopressor therapy	0.015*	8.358	1.508-46.332
Septic shock at MICU admission	0.503	1.553	0.428-5.632

## Discussion

A typical patient with S-AKI receiving CRRT was a middle-aged to older male admitted to the MICU from a medical ward who required respiratory, hemodynamic, and renal support. Similarly, sepsis was more common in male patients in a large study by Campanelli et al. (498.146 patients), where men accounted for 54-61% of cases [14]. Existing sex differences in infection susceptibility may be explained by endocrine-immunological interactions and the suppressive effects of androgens on cell-mediated immune responses [15].

The previously mentioned AKI-EPI study highlighted several important findings: AKI is very common in ICU settings (nearly 57% of ICU patients develop AKI within the first week), sepsis is one of the most important risk factors, and patient outcomes are closely related to the severity of kidney dysfunction (patients with KDIGO stage III have a sevenfold higher mortality rate). Since all patients in the present study were septic and required CRRT (KDIGO stage III), they were considered at high risk of mortality, which is consistent with our findings.

The 28-day all-cause mortality rate was 69.8% and represents a notably high mortality rate. This finding may reflect the high proportion of patients admitted to the MICU with septic shock, multiorgan failure, and coexisting comorbidities. On the other hand, the result may be attributed to limited personnel capacity, difficulties in ensuring isolation measures, insufficient infection prevention measures, and antimicrobial resistance. Other similar studies reported lower mortality rates, including a study from Spain/United States (90-day mortality was 62.9% among patients with S-AKI and septic shock in the ICU), a study from South Korea (90-day mortality was 58.5% among patients with S-AKI), and a large study from the United States (overall mortality 25%; however, the presence of septic shock doubled the mortality rate) [9,16,17]. 

When analyzed separately, patients admitted to MICU with septic shock had a higher mortality rate compared with those presenting with sepsis without shock (77.8% vs. 54%). Disease severity, reflected by elevated SOFA and SAPS II scores, was observed in nonsurvivors (p< 0.001), and the SAPS II score was identified as one of the independent predictors of mortality in logistic regression analysis. The high rate of septic shock at MICU admission (and admissions from medical wards) underscores the need for healthcare personnel education on early sepsis recognition, timely and appropriate management, and prompt MICU consultation.

Nonsurvivors had a longer median hospital stay prior to MICU admission (two days vs. one day), but a shorter MICU length of stay (p= 0.003), potentially reflecting higher disease severity and mortality. However, the analysis of the Kaplan-Meier mortality curve showed a median duration of MICU survival of 12 days. Hypertension, diabetes, and cardiomyopathy were the most prevalent comorbidities and may therefore represent risk factors for S-AKI. A similar result was reported in the systematic review and meta-analysis by Liu et al., where the identified risk factors were hypertension, diabetes, chronic kidney disease, cardiovascular disease, and liver disease [18]. The surprisingly high proportion of patients with malignancies (16.7%) may be related to the effects of various oncological treatments, as well as the high prevalence of these diseases. All patients showed abnormal laboratory findings at CRRT initiation, including inflammatory markers, urea, creatinine, D-dimer, and hemoglobin, with statistically significant differences in albumin, troponin I, and lactate among nonsurvivors.

Hypoalbuminemia was identified as an independent predictor of mortality in S-AKI patients, and may be explained by severe inflammation, increased vascular permeability, catabolism during critical illness, and the impact of underlying comorbidities [19,20]. Elevated troponin I and hyperlactatemia may reflect inflammation-induced ischemia and hypoperfusion, resulting in septic cardiomyopathy and metabolic disturbances, which are further exacerbated by S-AKI [21-24].

Nonsurvivors had multiorgan failure, including respiratory failure requiring invasive mechanical ventilation, and hemodynamic instability necessitating vasopressors (p< 0.001), which is consistent with previous reports [25,26]. The need for vasopressor therapy was also an independent predictor of mortality in S-AKI patients. Intermittent hemodialysis requires patient transfer to another location and does not provide prolonged renal support (median session duration of four to five hours; typically once daily). Therefore, following appropriate personnel training, CRRT is the preferred renal supportive therapy in our center. CVVHDF mode was used in all patients in accordance with the local MICU protocol for septic patients, with heparin as the predominant anticoagulant, mainly due to citrate shortages in that period. Oliguria/anuria with consequent volume overload and severe metabolic acidosis were the primary indications for CRRT, either alone or in combination. Although there is a lack of guidelines for initiating CRRT, these indications are recognized in the literature as absolute due to their urgency [27].

For all patients, CRRT was initiated on the first day of MICU stay, and each patient received one to two CRRT sets. Hemoadsorption represents an important component of the adjunctive treatment in sepsis and septic shock [28]; however, current evidence remains insufficient to support definitive recommendations regarding the use of blood purification techniques in sepsis management. Hemoadsorption was used as a treatment option in 39.58% of patients, more frequently among nonsurvivors (60.5%), and did not affect patients' outcomes. Clinical decision to use a hemoadsorption filter was made in the presence of a combination of hemodynamic instability, high vasopressor requirements, and elevated inflammatory markers. The most commonly used filter was CytoSorb (31.3%), which was partly selected based on technical availability. Although it did not affect outcomes in our cohort, hemoadsorption is an important immunomodulatory therapy in hyperinflammatory states with multiorgan failure [29].

The most prevalent sources of sepsis were pneumonia, urinary tract infection, multiple-site infection, and the abdomen. In the study of Jegenathan et al., patients with pulmonary, unknown, or multiple sources of sepsis had the highest rates of multiorgan failure and the highest hospital mortality [30], similar to our study. At MICU admission, biological samples for microbiological analysis were obtained from all patients when possible. Blood cultures were positive in 35.42% of patients (domination of gram-positive bacteria, 21.87%), urine cultures were positive in 19.79% of patients (domination of gram-negative bacteria, 12.50%), and tracheal aspirate/BAL were positive in 36.45% of patients (domination of gram-negative bacteria, 27.08%). A significant difference in distribution of tracheobronchal isolates was observed between survivors and nonsurvivors (p= 0.002), suggesting an association between mortality and pathogen type, and as expected, the majority of positive isolates were gram-negative bacteria. A high proportion of negative microbial cultures could be explained by prior antibiotic therapy and sampling-related limitations. Among the patients, 10 had a concurrent viral infection, six had candidiasis, and three had aspergillosis. It is well established that gram-negative microorganisms cause sepsis more frequently than gram-positive, and that inflammatory markers (CRP, PCT, tumor necrosis factor alpha) are higher in sepsis caused by gram-negative bacteria [31]. 

An important strength of this study lies in its originality and its setting in Bosnia and Herzegovina. However, the study also has several limitations, including a small sample size, a retrospective, single-center design, short follow-up, potential selection bias, and incomplete microbiology data. The additional research involving larger, prospective, and multicenter studies is necessary to reach more definitive conclusions.

## Conclusions

Patients with S-AKI requiring CRRT represent a high-risk subgroup, particularly when presenting with septic shock. Independent predictors in this LRS MICU setting included high SAPS II scores, vasopressor requirement, and hypoalbuminemia at CRRT initiation. Promotion of early recognition and management of sepsis, along with more rigorous implementation of hospital infection prevention measures are essential to improve outcomes. Furthermore, multicenter collaboration in LRS is needed to validate these findings and share experiences in similar settings. 
